# Characteristics and Adoption Success of Shelter Dogs Assessed as Resource Guarders

**DOI:** 10.3390/ani9110982

**Published:** 2019-11-17

**Authors:** Betty McGuire

**Affiliations:** Department of Ecology and Evolutionary Biology, Cornell University, Ithaca, NY 14853, USA; bam65@cornell.edu

**Keywords:** dog, food aggression, food guarding, resource guarding, shelter, behavior, adoption, return rate

## Abstract

**Simple Summary:**

Dogs that aggressively guard resources, such as food, toys, and sleeping sites, can pose risk to people unfamiliar with canine communication. Such dogs also present challenges to animal shelters, which typically screen for food-related guarding during behavioral evaluations. Some shelters euthanize dogs that aggressively guard food, whereas others restrict adoptions. However, few studies have examined the characteristics and adoption success of dogs that guard food in shelters. I analyzed demographic data and adoption success of dogs assessed as resource guarders at a shelter in New York (NY) over a nearly five-year period. Fifteen percent of the dog population was identified as resource guarders during shelter behavioral evaluations. Resource guarding was more common in adults and seniors than in juveniles, and it was more common in small and large dogs than medium-sized dogs. While spayed females were more likely than intact females to guard food, neutered males and intact males did not differ in their propensity to guard food. Dogs that showed severe guarding were more likely to be returned by adopters, but almost all were successfully re-adopted. These findings provide a detailed description of food guarders in a shelter dog population and show that most such dogs were successfully re-homed.

**Abstract:**

Some domestic dogs aggressively guard resources. Canine resource guarding impacts public health through dog bites and affects dog welfare through adoption and euthanasia policies at animal shelters. However, little is known about the demographic characteristics and adoption success of dogs assessed as resource guarders during shelter behavioral evaluations. I reviewed nearly five years of records from a New York (NY) SPCA and categorized 1016 dogs by sex; age; size; reproductive status; and resource guarding. I then examined how these characteristics influenced the returns of dogs by adopters. The prevalence of resource guarding in this shelter dog population was 15%. Resource guarding was more common in adult and senior dogs than in juvenile dogs; and it was more common in small and large dogs than medium-sized dogs. Spayed females were more likely than intact females to guard food; neutered males and intact males did not differ in their likelihood of food guarding. Most dogs identified as resource guarders showed mild to moderate guarding. Severe guarders were more likely to be returned by adopters; although almost all were eventually re-adopted and not returned to the shelter. Data presented here provide the most comprehensive description of resource guarders in a shelter dog population and show the successful re-homing of most.

## 1. Introduction

Some domestic dogs are possessive of resources such as food, toys, and sleeping sites, and they display threatening or aggressive behavior when a person approaches or attempts to take control of the resource. Such resource guarding occurs in homes and in animal shelters. One survey of animal shelters in the United States found that most shelters test for food guarding as part of their canine behavioral evaluations, and about half do not make available for adoption dogs assessed as food aggressive [1]. Shelters that make food guarding dogs available for adoption often place restrictions on who can adopt them (e.g., experienced dog owners with no young children in the household), which can prolong the time these dogs remain in shelters [2,3]. Thus, canine resource guarding can impact not only public health through dog bites, which are the most extreme form of guarding behavior [4], but also dog welfare via shelter policies on adoption and euthanasia [1,2,3]. The need for informed re-evaluation of shelter policies whereby all dogs classified as food aggressive are euthanized is especially critical given evidence that dogs assessed as food aggressive in a shelter do not necessarily guard food in their adoptive home [1,3]. In addition to these findings specific to food aggression and the predictive utility of behavioral evaluations [1,3], other research has more generally revealed the inadequacies of behavioral evaluations [5,6,7].

Few studies have examined characteristics of dogs that guard food. Most such studies have been based on owner reports of food guarding by dogs in the home [4,8,9] rather than observations of dogs during behavioral evaluations at shelters; one study used both shelter evaluations and reports from adopters [3]. For studies based on owner reports, two found that mixed breed dogs were more likely than purebred dogs to guard food [4,8]. Owner reports also identified increasing age of dog at acquisition as a predictor of food guarding [8]. A dog’s body size, as estimated by height at withers, was found to be negatively correlated with owner-directed aggression, a category that included resource guarding [9]. Conflicting results have been obtained regarding the influence of the sex of dogs on the likelihood of resource guarding. One study, based on owner reports, indicated that males were more likely than females to guard resources, and this was particularly true for neutered males [4]. In contrast, Marder et al. [3] found no sex difference in incidence of food aggression based on either shelter behavioral evaluations or subsequent reports by adopters. To date, no study has examined multiple demographic characteristics of dogs assessed as displaying food aggression during behavioral evaluations at shelters; current information is limited to one study that examined the influence of a single characteristic, the sex of shelter dogs, on the likelihood of food guarding during behavioral testing [3]. Understanding the characteristics associated with the expression of food guarding could serve as the basis for future research on the causation of guarding behavior [4]. Additionally, given that some shelters do not behaviorally evaluate all dogs made available for adoption [2], information on additional characteristics that might be associated with food aggression, such as age, reproductive status, and body size, could be useful to staff making decisions concerning which dogs to evaluate.

Two studies have examined the adoption success of dogs assessed as food aggressive in shelters. Mohan-Gibbons et al. [1] identified 96 food aggressive dogs at one shelter, placed them on a behavior modification program, and contacted their adopters three times in the months following adoption (adopters were asked to continue the behavior modification program that had begun in the shelter). Marder et al. [3] followed 97 shelter dogs, some of which were food aggressive and others not, and contacted adopters at least three months after adoption. Both studies found that dogs assessed as food aggressive during shelter behavioral evaluations did not necessarily guard food in their new homes, although the percentages of adopted dogs that continued food guarding in the home varied considerably, ranging from less than 10% [1] to 55% [3]. Results from both studies indicated that even if dogs displayed food aggression in the home, adopters did not consider the behavior to be a major challenge [1,3]. Mohan-Gibbons et al. [1] also found that the return rate for dogs assessed as food aggressive at their study shelter was lower than that for dogs assessed as not food aggressive; Marder et al. [3] did not provide return rates. No study has examined how food guarding, when considered with demographic characteristics such as sex, age, and body size, influences return rates. Additionally, no study has examined how the severity of food aggression (mild to moderate versus severe) influences return rates.

To further inform shelter policies regarding resource guarding dogs, additional information is needed on the demographic characteristics and adoption success of dogs identified as resource guarders during shelter behavioral evaluations. This paper considers food-related guarding; it does not consider other forms of resource guarding, such as the guarding of toys or sleeping sites. I reviewed nearly five years of records from a New York (NY) SPCA to develop a demographic profile for dogs assessed as resource guarders at the shelter and to determine the success of these dogs once adopted. I first examined whether sex, age class, reproductive status (intact versus spayed or neutered), or body size could be used to predict resource guarding, and then I assessed how these demographic characteristics, along with resource guarding, influenced the returns of dogs by adopters.

I predicted that likelihood of resource guarding would increase with the age of dogs, given the association found between food guarding and increasing age of dogs at acquisition [8]. Based on data indicating that behavioral problems are more common in small dogs than in large dogs [9], I predicted that small dogs would be more likely than medium and large dogs to display guarding behavior. I did not expect likelihood of resource guarding to vary by sex or reproductive status, given findings of no sex difference in the incidence of food guarding during shelter evaluations [3] and little or no effect of gonadectomy on aggression directed by dogs to people [10]. Adopters often cite behavioral problems as their reason for returning dogs to shelters [11,12,13,14], so I predicted that dogs assessed as resource guarders in the shelter would have higher return rates than dogs assessed as non-resource guarders and that severe resource guarders would be returned more frequently than dogs that showed mild to moderate guarding or no guarding.

## 2. Materials and Methods 

I analyzed records of dogs at the Tompkins County SPCA in Ithaca, NY, USA. These records included data input by shelter staff into the PetPoint data management system from 1 September 2014 through 31 May 2019. Records included information on dog intakes (including returns), behavioral evaluations, and adoptions (*n* = 1016 adopted dogs; puppies excluded, see Section 2.2). Tompkins County SPCA is a no-kill, open-admission shelter with scheduled intake. The shelter has a small set of dog foster parents and allows for overnight fostering with dog volunteers. Additional programs to promote dog adoptability include: volunteer dog walking, volunteer in-kennel companionship, volunteer day-trips with dogs, playgroups for suitable pairs of dogs, nightly stuffed Kong enrichment, adoption promotion in local print and social media, off-site events to advertise dogs, and a volunteer group independently promoting dogs that are hard-to-place or have been at the shelter a long time. All procedures were carried out under protocol 2012-0150, which was approved by Cornell University’s Institutional Animal Care and Use Committee.

### 2.1. Dogs and Housing

Original sources were available for 1015 of the 1016 adopted dogs whose records I reviewed: owner surrendered, 473 (46.6%); transferred from other shelters, 343 (33.8%); picked up as strays, 166 (16.4%); and seized by animal control officers, 33 (3.2%). Most dogs at the Tompkins shelter were mixed breeds; the number of purebred dogs was unknown due to lack of pedigrees and DNA analyses. A brief description of housing and care of dogs is provided here because details have been presented elsewhere [15].

At intake, dogs were housed in the rescue building in chain link cages with an indoor space (2.2 m^2^) and outdoor run (3.5 m^2^). Veterinary staff examined each dog at intake or within a few hours of intake and performed vaccinations, flea control, fecal exam, deworming, and a heartworm test. Following the veterinary exam, each dog was scheduled for behavioral evaluation (see Section 2.2). Within a few days of the completion of the behavioral evaluation, dogs were moved to the pet adoption center, adjacent to the rescue building. Once on the adoption floor, dogs were housed in one of 13 cubicles, which ranged in size from 5.2 to 7.3 m^2^. Almost all dogs were housed individually; only dogs that came in together and staff deemed needed to stay together shared the same cubicle. Each cubicle contained a water bowl, a raised bed, a blanket, and a toy. Volunteers or staff either walked the dogs or brought them to an outdoor enclosure several times a day. Staff fed the dogs each day between 08:00 and 09:00 h and again between 15:00 and 16:00 h. Intact dogs were spayed or neutered when housed in either the rescue building or the pet adoption center; all dogs were spayed or neutered before adoption.

### 2.2. Behavioral Evaluations

Shelter staff evaluated each dog’s behavior using a series of tests based on Sternberg’s Assess-a-Pet [16], with modifications described by Bollen and Horowitz [17]; these modifications were made as part of the shelter’s standard operating procedures and were in place well in advance of the present study. Behavioral evaluations were performed approximately 3 days after intake and included nine tests in the following sequence: cage presentation; sociability; teeth exam; handling; arousal; food bowl (using a mix of kibble and canned food); possession (using a raw hide chew, pig ear, etc.); human stranger; and dog-to-dog. Behavioral responses on the food bowl test were organized into seven levels, listed in order of increasing intensity of response: (1) stopped eating and backed away from the dish; (2) continued eating without showing any signs of uneasiness; (3) moved muzzle deeper into the dish and ate faster; (4) stiffened slightly; (5) moved muzzle toward the Assess-a-Hand; (6) stiffened, exhibited whale eye, and snarled; and (7) froze, growled, lunged, snapped, and bit the Assess-a-Hand. Behavioral responses on the possession test were organized into five levels, also listed in order of increasing intensity of response: (1) readily dropped the item; (2) allowed the Assess-a-Hand to take the item; (3) resisted letting go of the item but did not show outward aggression; (4) stiffened, exhibited whale eye, and snarled; and (5) froze, growled, lunged, snapped, and bit the Assess-a-Hand. When a dog was very uncomfortable with the Assess-a-Hand, the evaluator used her own hand to remove the food bowl and chew. Dogs were assessed as resource guarders if they exhibited at least one of the following behaviors during either the food bowl test, possession test, or both tests: stiffened, exhibited whale eye, snarled, froze, growled, lunged, snapped, or bit the Assess-a-Hand. For one analysis, I classified resource guarding dogs as exhibiting either mild to moderate resource guarding (stiffened, exhibited whale eye, snarled, froze, or growled) or severe resource guarding (lunged, snapped, or bit the Assess-a-Hand) during either the food bowl test, possession test, or both tests. This categorization was based on that described by Mohan-Gibbons et al. [2].

Though the behavior of puppies was formally evaluated by staff, the tests differed somewhat from those of older dogs (e.g., recent tests were conducted in the cubicle in which puppies were housed on the adoption floor rather than in the conference room where tests were conducted for dogs in older age classes). Additionally, puppy results were not input into the PetPoint database. For these reasons, puppies were not included in the present study.

### 2.3. Statistical Analyses

I classified dogs by sex, age class, body size, and reproductive status. The ages of dogs were estimated by shelter veterinarians. For the purpose of this study, the following age classes were defined based on those used in previous studies [18,19]: juveniles, from 4 months to <1 year; adults, from 1 year to <8 years; and seniors, ≥8 years. The number of dogs in each sex and age class during the study period was as follows: males, 100 juveniles, 348 adults, 66 seniors; females, 99 juveniles, 340 adults, and 63 seniors. I used the body mass recorded at veterinary intake exams to classify adult and senior dogs into the following size classes: small, <11 kg; medium, 11–24 kg, and large, ≥25 kg (categories modified from those used by Taylor et al. [20]). I did not assign juveniles a size class because they were still growing; thus, juveniles were excluded from data analyses in which body size was a variable. Mature dogs (adults and seniors) fell into the following size classes: small, 32.1%; medium, 37.7%; and large, 30.2% (body mass was not available for one adult female out of the combined 817 adults and seniors). The following percentages of dogs by sex and age class were intact at the time of behavioral evaluation: males, 83.0% of juveniles, 53.7% of adults, 30.3% of seniors; females, 85.9% of juveniles, 54.4% of adults, and 25.4% of seniors. The final dispositions of returned dogs were classified as adopted again and not returned, euthanized for either behavioral or medical reasons, transferred to a rescue group, or returned to the original owner (i.e., the person who originally surrendered the dog to the shelter experienced a change in living situation such that he or she was able to take the dog back). The final dispositions of dogs returned toward the end of the study period (May 2019) were followed for an additional 4 months.

I used logistic regression to determine significant predictors of resource guarding. Fixed factors in the first model for resource guarding were sex, reproductive status, and age class (juveniles, adults, and seniors). I then excluded juveniles from the data set so that body size could be added as a fixed factor in the second model for resource guarding. I also used logistic regression to determine significant predictors of a dog being returned to the shelter by adopters. Fixed factors in the first model for likelihood of return were sex, age class (juveniles, adults, and seniors), and resource guarding status. In the second model for likelihood of return, I excluded juveniles from the data set so that body size could be added as a fixed factor. Finally, in the third model for likelihood of return, I considered the level of resource guarding and categorized dogs as non-resource guarders, mild to moderate resource guarders, or severe resource guarders, as defined in Section 2.2. For all models, I examined the main effects and two-way interactions; I dropped two-way interactions that were not significant from final models. All dogs were spayed or neutered prior to adoption, so reproductive status was not a fixed factor in any of the models for likelihood of return. Statistical analyses were completed in JMP Pro (version 13.1.0).

## 3. Results

### 3.1. Resource Guarding

Over the nearly five-year study period, staff evaluated the behavior of 1051 individual dogs (juveniles, adults, and seniors); 161 dogs were assessed as resource guarders, resulting in a prevalence of 15.3% of dogs evaluated. Fifteen of the resource guarding dogs were not made available for adoption: 10 were euthanized for behavioral reasons and one for medical reasons; three were transferred to rescue groups; and one was returned to her original owner. All of the results that follow pertain to the 1016 dogs that were behaviorally evaluated and made available for adoption.

Overall, 14.4% of dogs moved to the adoption floor were classified as resource guarders based on behavioral evaluations (146/1016; juveniles, adults, and seniors). Of the dogs assessed as resource guarders, 30.8% (45/146) guarded on the food bowl test, 83.6% (122/146) guarded on the possession test, and 17.1% (25/146) guarded on both tests. On both the food bowl test and the possession test, freezing was the most common behavior displayed by resource guarding dogs, and lunging was the least common (Table 1). The two most extreme behaviors, snapping and biting the Assess-a-Hand, occurred in less than 14% of resource guarding dogs (Table 1).

The percentages of dogs assessed as resource guarders in relation to main effects of sex, age class, reproductive status, and body size are shown in Table 2. Age class was a significant predictor of resource guarding (*X*^2^ = 13.53, *d.f.* = 2, *p* = 0.001), with adults and seniors more likely than juveniles to show guarding behavior (Table 2, second column). Seniors tended to be more likely than adults to guard resources (*p* = 0.08; Table 2, second column). There was a significant sex by reproductive status interaction for likelihood of resource guarding (*X*^2^ = 5.24, *d.f.* = 1, *p* = 0.022). While spayed females (17.1%; 37/216) were more likely than intact females (9.1%; 26/286) to guard food, neutered males (15.2%; 34/224) and intact males (16.9%; 49/290) did not differ in their propensity to guard food. Neutered males and intact males also were more likely than intact females to guard food.

When juveniles were excluded from the data set to allow for the inclusion of body size as a fixed factor in the model, body size was a significant predictor of resource guarding (*X*^2^ = 7.05, *d.f.* = 2, *p* = 0.03), with small dogs and large dogs more likely than medium dogs to display guarding (Table 2, third column). Small and large dogs did not differ from one another in propensity to guard. With juveniles excluded, age class did not predict food guarding (*X*^2^ = 1.63, *d.f.* = 1, *p* = 0.20; Table 2, third column). As before, there was a significant sex by reproductive status interaction for likelihood of food guarding (*X*^2^ = 5.45, *d.f.* = 1, *p* = 0.02). While spayed females (18.3%; 37/202) were more likely than intact females (10.4%; 21/201) to guard food, neutered males (15.5%; 32/207) and intact males (20.3%; 42/207) did not differ in their propensity to guard food. Intact males were more likely than intact females to guard food.

### 3.2. Returns of Dogs by Adopters

Of the 1016 dogs adopted during the nearly five-year study period (juveniles, adults, and seniors), 181 (17.8%) were returned to the shelter at least once. The number of returns per dog ranged from one to six, with one being most common: one return, 80.7% (146/181); two returns, 17.1% (31/181); three returns, 1.7% (3/181); and six returns, 0.5% (1/181). The percentages of adopted dogs returned to the shelter in relation to main effects of sex, age class, resource guarding status, and body size are shown in Table 3. Age class did not predict likelihood of return (*X*^2^ = 2.94, *d.f.* = 2, *p* = 0.23; Table 3, second column). There was a borderline significant sex by resource guarding status interaction for likelihood of return (*X*^2^ = 3.80, *d.f.* = 1, *p* = 0.0514). While food aggressive males (27.7%; 23/83) were more likely than non-food aggressive males (17.2%; 74/431) to be returned, food aggressive females (14.3%; 9/63) and non-food aggressive females (17.5%; 77/439) did not differ in their likelihood of return. Food aggressive males also were more likely to be returned than food aggressive females and non-food aggressive females.

Body size was a significant predictor of a dog being returned (*X*^2^ = 15.38, *d.f.* = 2, *p* = 0.0005), with large dogs more likely than small and medium dogs to be returned (Table 3, third column; juveniles excluded). Small and medium dogs did not differ from one another in their likelihood of return. Age class did not predict likelihood of return when the data set was restricted to adults and seniors (*X*^2^ = 0.76, *d.f.* = 1, *p* = 0.38; Table 3, third column). As before, logistic regression revealed a sex by resource guarding status interaction for likelihood of return (*X*^2^ = 4.47, *d.f.* = 1, *p* = 0.034). While food aggressive males (27.0%; 20/74) were more likely than non-food aggressive males (17.9%; 61/340) to be returned, food aggressive females (13.8%; 8/58) and non-food aggressive females (19.1%; 66/345) did not differ in their likelihood of return. Note, however, that the subsequent pairwise comparison between percentages of food aggressive males and non-food aggressive males returned by adopters fell short of statistical significance (*p* = 0.08). There was a tendency for food aggressive males to be more likely than food aggressive females to be returned (*p* = 0.06). The interaction between body size and resource guarding status was not significant (*X*^2^ = 0.74, *d.f.* = 2, *p* = 0.69), indicating that returns of food aggressive dogs did not vary by size of dog.

Of the 146 dogs assessed as resource guarders at the shelter, 121 (82.9%) showed mild to moderate guarding, and 25 (17.1%) showed severe guarding. (Note: 25 does not equal the sum of number of dogs shown in Table 1 that lunged, snapped, and bit the Assess-a-Hand, because some dogs exhibited more than one of these behaviors during either the food bowl test, possession test, or both tests). The 25 dogs that showed severe guarding included two juveniles, 18 adults, and five seniors. Given the small numbers of juveniles and seniors in the severe guarding group, I did not include age class as a fixed factor in the third model for likelihood of return. When resource guarding was differentiated by level, guarding was a significant predictor of a dog being returned (*X*^2^ = 6.72, *d.f.* = 2, *p* = 0.035), with severe guarders more likely to be returned (40.0%; 10/25) than mild to moderate guarders (18.2%; 22/121) and dogs classified as non-resource guarders (17.5%; 152/870). Dogs showing mild to moderate guarding and dogs classified as non-resource guarders did not differ from one another in likelihood of return. Sex did not predict likelihood of return when resource guarding was differentiated by level (*X*^2^ = 0.40, *d.f.* = 1, *p* = 0.53).

Fifteen of the 25 dogs that exhibited severe guarding during behavioral evaluations were adopted and not returned to the shelter. For the remaining 10 dogs in the severe group, seven were returned once and then adopted without return; one was returned twice and then adopted without return; one was returned three times and then adopted without return; and one was returned twice and euthanized (this dog bit an adult in its second adoptive home). Thus, of the 25 dogs classified as severe resource guarders at the shelter, 24 (96%) were eventually placed in a home and not returned to the shelter.

The canine surrender profile form of the Tompkins shelter includes the statement, “Please explain why you need to relinquish your dog in your own words.” Reasons given for returns of the nine severe guarders that were eventually successfully re-homed included elimination in the house, owner allergies, unforeseen personal reasons, moving, aggression directed at another dog in the home, and over-arousal; one small dog bit the adopter’s grandson. None of the adopters completing the form described aggression around food; one adopter, who chose to provide a lengthy written explanation rather than completing the surrender form, described over-excitement around food, but stated the reason for surrender was unforeseen personal reasons. The surrender form also includes the statement, “Please check all that apply to your dog’s personality” and lists the following options: friendly, shy, independent, fearful, playful, affectionate, aloof, aggressive, and overly reactive. Two adopters listed shy, fearful, and overly reactive; one listed aggressive but to another dog; some combination of friendly, independent, playful, and affectionate were checked by remaining adopters.

Of the 181 dogs returned during the study period, one was brought to a shelter located in a different state, and his final disposition was unknown. The final dispositions for the remaining 180 dogs returned at least once to the Tompkins shelter were as follows: re-adopted and not returned to the shelter, 87.2% (157/180); euthanized for either behavioral or medical reasons, 8.9% (16/180); transferred to a rescue group, 2.2% (4/180); and returned to the original owner, 1.7% (3/180).

## 4. Discussion

Measures of prevalence and severity of resource guarding in dogs at the Tompkins County SPCA, as well as overall return rate, are similar to those reported previously for dogs at other shelters. Fifteen percent of dogs behaviorally evaluated at the Tompkins shelter were assessed as resource guarders. This measure of guarding prevalence is similar to those reported by Mohan-Gibbons et al. [1], who surveyed 77 shelters in the United States and found that percent of dog populations exhibiting food guarding ranged from 7–30%, with an average of 14%. In a later study involving nine shelters, Mohan-Gibbons et al. [2] reported that 17% of behaviorally evaluated dogs were classified as food guarders, and Marder et al. [3] found that 21% of dogs at one shelter exhibited aggression around food. Additionally, 83% of the dogs assessed as resource guarders at the Tompkins shelter showed mild to moderate guarding, and 17% showed severe guarding; these same percentages were obtained by Mohan-Gibbons et al. [2] for their sample of shelter dogs assessed as food guarders. Finally, the overall return rate at the Tompkins shelter (18%) was similar to the average return rate reported for shelters in the United States, United Kingdom, and Italy (15%; see review by Protopopova and Gunther [21]). The consistency in both the prevalence and degree of severity of resource guarding across shelters despite the use of different behavioral assessments (e.g., Assess-a-Pet, SAFER^TM^, blends and modifications of these and other assessments, as well as assessments developed by individual shelters) and the similarity in overall return rates suggest that the present findings on characteristics and returns of resource guarding dogs at the Tompkins shelter might generalize to other shelters.

Age class was a significant predictor of resource guarding in dogs at the Tompkins shelter, with adults and seniors more likely than juveniles to show food-related guarding during behavioral evaluations. Additionally, there was a tendency for seniors to be more likely than adults to guard food. This is the first study based on direct observations of dogs during behavioral evaluations at a shelter to examine the relationship between resource guarding and age. Using owner responses to a questionnaire distributed by an Australian dog magazine, McGreevy and Masters [8] reported that food-related aggression was associated with increasing age of dogs at acquisition (485 respondent households and a total of 690 dogs obtained from a variety of sources, including pet shops, breeders, pounds, shelters, friends, and family). Dogs included in the survey ranged from eight weeks to over 11 years old [22]; thus, this study differed from the present study not only in its method of obtaining information on dogs with respect to resource guarding but also in its inclusion of data on puppies (results from evaluations of puppies were not available in the present study). Guarding behaviors have been described in puppies only a few weeks old [23]. It would be useful in future studies with shelter dogs to include data from puppies to provide a more complete picture of age-related patterns in resource guarding.

Body size, based on body mass, was a significant predictor of resource guarding in the dog population at the Tompkins shelter, with small dogs and large dogs more likely than medium dogs to display guarding behavior. Small and large dogs did not differ from one another in propensity to guard resources. Using behavioral and body mass data collected from dog owners who completed the Canine Behavioral Assessment and Research Questionnaire (C-BARQ; 49 common breeds were represented in the study sample) and height data drawn from breed standards, McGreevy et al. [9] found that height was negatively correlated with owner-directed aggression, a category that included resource guarding. More specifically, McGreevy et al. [9] found that shorter dogs were more likely than taller dogs to display threatening or aggressive responses to household members in a variety of situations, which included being roughly handled, stared at, challenged, stepped over, or approached when possessing food or objects; body mass, however, did not predict owner-directed aggression. It is possible that absence of a relationship between body mass and owner-directed aggression in the study by McGreevy et al. [9] reflected the same pattern found here, i.e., despite differing from medium dogs, small (light) and large (heavy) dogs did not differ from one another in their tendency to guard resources. However, direct comparison of the present results with those of McGreevy et al. [9] are difficult given major differences between the two studies in methods of classifying dogs with respect to guarding behavior (direct observations by shelter staff during behavioral evaluations versus owner reports), dog populations (primarily mixed breed dogs at a shelter versus purebred dogs in homes), and scope of behavioral categories (restricted to food-related guarding versus owner-directed aggression, which included resource guarding and several other situations involving dogs and household members). Nevertheless, the present finding that small dogs were more likely than medium dogs to show resource guarding is consistent with the general pattern that problem behaviors are more common in small dogs [9]. Factors underlying the present finding that large dogs were more likely than medium dogs to guard resources remain to be determined.

The effect of reproductive status on propensity to guard resources varied by sex at the Tompkins shelter, with spayed females more likely to guard than intact females, and no difference in guarding propensity between neutered males and intact males. Neutered males and intact males were more likely to guard than intact females and did not differ from spayed females. To my knowledge, the present study is the first based on shelter behavioral evaluations to examine the relationship between resource guarding and both sex and reproductive status in dogs. The present findings differ from those of Jacobs et al. [4], who surveyed dog owners and found that dogs showing aggressive resource guarding in the home were more likely to be male and neutered. Jacobs et al. [4] acknowledged that dogs in their study might have been neutered after showing resource guarding aggression, in which case neutering might be considered a consequence of aggression rather than a cause (age at castration and age at first display of resource guarding aggression were not obtained from owners). The present findings are consistent with the general conclusions of Farhoody et al. [10] that gonadectomy does not result in predictable decreases in aggression in all male and female dogs.

Initial analyses that coded dogs at the Tompkins shelter as either resource guarders or non-resource guarders indicated that the effect of resource guarding on likelihood of a dog being returned to the shelter varied by sex. More specifically, whereas food aggressive males were more likely to be returned than non-food aggressive males, food aggressive females and non-food aggressive females did not differ in their likelihood of return. In other words, food aggression either increased returns (in the case of males) or had no effect on returns (in the case of females). The only other data available comparing return rates of food aggressive and non-food aggressive dogs to shelters are those of Mohan-Gibbons et al. [1], who reported slightly lower return rates for dogs identified as food aggressive (5%) when compared to dogs assessed as not food aggressive at one shelter (9%); the sex of dogs was not considered with resource guarding. Possible explanations for these different patterns include the following aspects of the study design used by Mohan-Gibbons et al. [1], which differ from the present study: pit bulls and Rottweilers were excluded, the inclusion criteria focused on dogs showing highly adoptable behavior except on the food bowl test, and food aggressive dogs were in a behavior modification program while in the shelter and later in their adoptive home (although many adopters did not comply). Finally, the study by Mohan-Gibbons et al. [1] used results from the food bowl test, whereas results from the food bowl test and possession test were used here.

When resource guarding was differentiated by level of severity in the present study, guarding was a significant predictor of a dog being returned, with severe resource guarders more likely to be returned than mild to moderate guarders and dogs classified as non-guarders. Dogs showing mild to moderate guarding did not differ from dogs classified as non-guarders in their likelihood of return. The reasons for return of dogs identified in the shelter as severe guarders typically did not involve aggression to humans; instead, the reasons given were those commonly provided by adopters returning dogs to shelters (e.g., allergies, moving, personal reasons, not getting along with other pets, and behavioral problems such as elimination in the house and over-arousal; [13,24]). Importantly, despite the greater likelihood of return of severe resource guarding dogs to the shelter, almost all of these dogs (24 of 25) were eventually placed in a home. Adopter surveys have revealed that many dogs assessed as food aggressive in shelters do not guard food in their adoptive homes, and, even when dogs continue to display food guarding in the home, adopters do not consider it to be a major problem [1,3]. Taken together, the present results on adoption success and published results from adopter surveys [1,3] strongly suggest that shelter staff consider adoption rather than euthanasia for most dogs identified as resource guarders during behavioral evaluations in shelters.

Body size also influenced likelihood of return at the Tompkins shelter. Using body mass as the measure of body size, I found that large dogs were more likely to be returned than small and medium dogs. Similar results have been obtained by Marston et al. [12], Diesel et al. [14], and Posage et al. [25]; suggested explanations for the observed pattern include the greater costs, space needs, and exercise requirements of large dogs, as well as the increased challenges of managing any behavioral issues. Interestingly, in the present study, the body size by resource guarding status interaction was not significant in the analysis of factors affecting likelihood of return, indicating that returns of food aggressive dogs to the Tompkins shelter did not vary by size of dog (e.g., adopters were not more likely to return large food aggressive dogs than small food aggressive dogs).

Most dogs (87%) returned to the Tompkins shelter were subsequently re-adopted and not returned to the shelter; 9% of returned dogs were euthanized, and the remaining 4% of dogs were either transferred to a rescue organization or returned to the original owner. Lower rates of re-adoption and higher rates of euthanasia have been noted for returned dogs at other shelters. Patronek et al. [26] reported that 50% of returned dogs were subsequently adopted; these authors also found a 33% euthanasia rate for all potentially adoptable dogs at the study shelter, although this value likely represented an upper limit (the percentage of returned dogs euthanized was not described). Across three Australian shelters, Marston et al. [12] reported 57% of returned dogs were subsequently re-adopted, 38% were euthanized, and fates were unknown for the remaining 5%. I cannot definitively state that all re-adopted dogs remained in the home; I can only state that the dogs were not returned to the Tompkins shelter. However, several policies at the Tompkins shelter encourage people who do not wish to keep their adopted dog to return it to the shelter rather than give the dog to someone else. First, all adopters must sign a contract stating that they will return the dog to the Tompkins shelter if the dog is not a good fit for their household. Second, all dogs receive a microchip, which is registered before leaving the shelter, so dogs can be identified if brought elsewhere, such as to a different shelter. Finally, if a dog is returned within two weeks of adoption, then the shelter refunds 75% of the adoption fee. For these reasons, I expect that most, if not all, re-adopted dogs remained in their new homes.

## 5. Conclusions

The prevalence of resource guarding during behavioral evaluations was 15% in the population of dogs at the Tompkins shelter, which is comparable to that observed at other shelters in the United States [1]. The demographic profile developed for dogs identified as resource guarders at the Tompkins shelter indicated they were more likely to be adults and seniors than juveniles, and when fully grown, more likely to be either small or large than medium with respect to body size based on body mass. Spayed females, intact males, and neutered males were more likely than intact females to guard resources. Ideally, shelters should conduct behavioral evaluations of all dogs made available for adoption. However, some shelters do not follow this procedure, especially with dogs considered highly adoptable at intake [2]. The profile provided here may help such shelters make informed decisions about which dogs should be evaluated for resource guarding. For example, shelters might be less likely to assess small dogs than large dogs, but the data presented here show that small dogs are just as likely as large dogs to display food-related guarding during behavioral evaluations, and those that do are just as likely as large dogs assessed as food guarders to be returned by adopters. The ability to generalize results presented here to other shelters will depend on how similar other shelters are to the Tompkins shelter with respect to dog populations and shelter policies.

Most dogs assessed as resource guarders at the Tompkins shelter showed mild to moderate guarding. Dogs assessed as severe guarders were more likely to be returned by adopters than dogs assessed as mild to moderate guarders or non-guarders. However, almost all severe guarders that were returned to the shelter were eventually re-adopted and not returned. Thus, results from this population of shelter dogs indicate that most dogs identified as resource guarders during behavioral evaluations can be successfully re-homed, although it might take more than one effort at adoption. These data on adoption success, together with data showing that dogs assessed as food aggressive at shelters do not necessarily display food-related guarding in their adoptive homes [1,3], strongly suggest that shelter staff consider adoption rather than euthanasia for most dogs identified as resource guarders during behavioral evaluations in shelters.

## Figures and Tables

**Table 1:** The percentages of resource guarding dogs that displayed specific behaviors during the food bowl test and possession test. The number of dogs that displayed the behavior/number of dogs assessed as resource guarding on the particular test are in parentheses.

Behavior Shown ^1^	Food Bowl Test	Possession Test
Stiffened	20.0 (9/45)	32.8 (40/122)
Exhibited whale eye	20.0 (9/45)	9.8 (12/122)
Snarled	17.8 (8/45)	18.9 (23/122)
Froze	57.8 (26/45)	53.3 (65/122)
Growled	35.6 (16/45)	24.6 (30/122)
Lunged	0.0 (0/45)	4.9 (6/122)
Snapped	11.1 (5/45)	11.5 (14/122)
Bit Assess-a-Hand	13.3 (6/45)	8.2 (10/122)

^1^ Mild to moderate resource guarding included the behaviors from stiffened through growled; severe resource guarding included the behaviors lunged, snapped, and bit the Assess-a-Hand.

**Table 2:** The percentages of dogs assessed as resource guarders in relation to sex, age class, reproductive status, and body size. The number of dogs assessed as resource guarders/number of dogs evaluated and made available for adoption shown in parentheses. Within columns and specific variables, values with different superscript letters are significantly different (*p* ≤ 0.05).

Variable	Juveniles, Adults, and Seniors ^1^	Adults and Seniors ^1^
Sex		
Male	16.1 (83/514)	17.9 (74/414)
Female	12.6 (63/502)	14.4 (58/403)
Age class		
Juvenile	7.0 (14/199) ^a^	
Adult	15.1 (104/688) ^b^	15.1 (104/688)
Senior	21.7 (28/129) ^b^	21.7 (28/129)
Reproductive status		
Intact	12.4 (71/574)	15.4 (63/408)
Spayed/neutered	16.1 (71/440)	16.9 (69/409)
Body size		
Small		19.8 (52/262) ^d^
Medium		11.0 (34/308) ^c^
Large		18.7 (46/246) ^d^

^1^ Age classes included in analyses.

**Table 3:** The percentages of adopted dogs returned to the shelter in relation to sex, age class, resource guarding status, and body size. The number of dogs returned/number of dogs adopted shown in parentheses. Within columns and specific variables, values with different superscript letters are significantly different (*p* ≤ 0.05).

Variable	Juveniles, Adults, and Seniors ^1^	Adults and Seniors ^1^
Sex		
Male	18.7 (96/514)	19.3 (80/414)
Female	17.1 (86/502)	18.4 (74/403)
Age class		
Juvenile	14.1 (28/199)	
Adult	18.5 (127/688)	18.5 (127/688)
Senior	20.9 (27/129)	20.9 (27/129)
Resource guarding		
Yes	21.2 (31/146)	20.5 (27/132)
No	17.4 (151/870)	18.5 (127/685)
Body size		
Small		13.7 (36/262) ^b^
Medium		16.9 (52/308) ^b^
Large		26.8 (66/246) ^a^

^1^ Age classes included in analyses.

## Supplementary Materials

Supplementary File 1
